# Difenoconazole Exposure Induces Retinoic Acid Signaling Dysregulation and Testicular Injury in Mice Testes

**DOI:** 10.3390/toxics11040328

**Published:** 2023-03-30

**Authors:** Xiangqin Zheng, Yuexin Wei, Jiadong Chen, Xia Wang, Dinggang Li, Chengjun Yu, Yifan Hong, Lianju Shen, Chunlan Long, Guanghui Wei, Shengde Wu

**Affiliations:** Chongqing Key Laboratory of Children Urogenital Development and Tissue Engineering, Chongqing 400014, China

**Keywords:** difenoconazole, reproductive toxicity, testis, retinoic acid signaling, GC-2 cell, apoptosis

## Abstract

Difenoconazole (DFZ) is a broad-spectrum triazole fungicide that is widely utilized in agriculture. Although DFZ has been demonstrated to induce reproductive toxicity in aquatic species, its toxic effects on the mammalian reproductive system have yet to be fully elucidated. In vivo, male mice were administered 0, 20 or 40 mg/kg/d of DFZ via oral gavage for 35 days. Consequently, DFZ significantly decreased testicular organ coefficient, sperm count and testosterone levels, augmented sperm malformation rates, and elicited histopathological alterations in testes. TUNEL assay showed increased apoptosis in testis. Western blotting results suggested abnormally high expression of the sperm meiosis-associated proteins STRA8 and SCP3. The concentrations of retinoic acid (RA), retinaldehyde (RE), and retinol (ROL) were increased in the testicular tissues of DFZ-treated groups. The mRNA expression level of genes implicated in RA synthesis significantly increased while genes involved in RA catabolism significantly decreased. In vitro, DFZ reduced cell viability and increased RA, RE, and ROL levels in GC-2 cells. Transcriptome analysis revealed a significant enrichment of numerous terms associated with the RA pathway and apoptosis. The qPCR experiment verified the transcriptome results. In conclusion, our results indicate that DFZ exposure can disrupt RA signaling pathway homeostasis, and induce testicular injury in mice testes.

## 1. Introduction

Fungicides are pervasively utilized; thus, they can easily accumulate in the environment and cause deleterious effects on both human health and environmental safety. Since the 1950s, triazole fungicides (TOFs) have been extensively employed to inhibit fungi in crops, such as fruit, vegetable, and flower crops [1]. Sales of triazole fungicides accounted for 5.5% of the global insecticide market and 21.2% of the fungicide market in 2014 [2]. Various studies have reported that fungicides can induce adverse impacts on non-target organisms. DFZ is one of the most typical TOFs, and it has a variety of bioactivities that can block lanosterol-14R-demethylase and inhibit the formation of fungus-derived sterols, thereby preventing the spread of fungus-related illnesses [3]. Compared to other TOFs, DFZ causes greater acute toxicity [4]. Although the use of DFZ can increase land productivity and reduce crop damage, the environmental and human health hazards of DFZ cannot be ignored.

Due to the wide application and high persistence of DFZ, it can be detected in soil [5], water [6], air [7], and agricultural crops [8]. It was reported that over 1% of soil samples from northern China had DFZ concentrations higher than 0.1 mg/kg [9], the concentration of DFZ in agricultural water in Thailand (Salakru, Nong Sua) was estimated to be 0.028 mg/L, and the concentration of DFZ in air samples from Tanjung Karang was 86.49 ng/m^−3^ [10]. A French total infant diet study showed that DFZ was detected in more than 5% of infant food samples [11]. The concentration of DFZ in many crops is higher than the acceptable daily intake (ADI) value [12].

Increasing evidence shows that DFZ has potential cumulative effects [13], and it can threaten human health through skin contact, consumption or inhalation [14,15,16]. DFZ can cause endocrine disruption, embryotoxicity, teratogenic effects, developmental toxicity, hepatotoxicity, and genotoxic effects in zebrafish [17,18,19,20], and even lead to cardiovascular toxicity [21]. DFZ can induce apoptosis in HepG2 cells [22] and induce oxidative DNA damage and mitochondria-mediated apoptosis in SH-SY5Y cells [23]. DFZ has been shown to cause reproductive toxicity in aquatic species [24] and plants [25]. Regrettably, there has been a dearth of studies examining the reproductive toxicity of DFZ in mammals.

Retinoic acid (RA), a metabolite of vitamin A, is irreplaceable for organ development in mammals [26]. It is well-known that RA is critical for the proliferation and differentiation of germ cells [27]. Furthermore, normal RA synthesis and metabolism in the reproductive system are important for promoting resistance to the adverse effects of environmental endocrine disruptors. Deficient or excessive RA levels can lead to abnormal testicular development or testicular injury, so maintaining the balance of RA is essential. In the mouse testis, RA participates in spermatogonia differentiation and spermatocyte meiosis initiation [28]. TOFs have been reported to interfere with internal RA metabolism and RA levels by inhibiting CYP450 enzymes in plants and animals, and have development toxicity to the organism. Nevertheless, it remains unclear whether exposure to DFZ can induce abnormalities in the RA pathway in testes.

The current study was designed to examine the correlation between DFZ exposure and testicular damage in male mice, and to further investigate the potential participation of the retinoic acid (RA) pathway in this process through in vivo and in vitro studies. Our findings shed light on the environmental risks of DFZ, broaden our understanding of the toxic effects of DFZ on the male reproductive system, and provide a theoretical basis for the safe and rational use of DFZ.

## 2. Materials and Methods

### 2.1. Animal Model and In Vivo Treatment

Thirty healthy male C57BL/6 mice (three weeks old, 8–9 g) were obtained from the Laboratory Animal Center of Chongqing Medical University. Thirty mice were maintained under SPF conditions (12 h/12 h diurnal cycle, humidity of 55 ± 5%, temperature of 25 ± 2 °C) and provided with ad libitum access to food and water. The mice were randomly divided into three groups (10 in each): low-dose group (D20), high-dose group (D40), and control group (Ctrl). Corn oil (Aladdin, C116023, Shanghai, China) was administered to the control group, while DFZ (CAS, 119446-68–3, purity ≥ 98%) dissolved in corn oil, 20 and 40 mg/kg/d, was orally administered to the D20 and D40 groups for 35 consecutive days, respectively. Twelve hours after administration ceased, the mice were weighed and then anesthetized with phenobarbital sodium. Blood samples were collected from each mouse into a separate tube in order to isolate the serum for further biochemical analysis. Finally, the mice were immediately sacrificed, and the epididymis and testes were harvested from both sides and weighed. Then, the testicular organ coefficient (testis weight/body weight) was calculated.

### 2.2. Sperm Count and Sperm Malformation Rate

The isolated epididymis tissues were placed in a sterile glass container containing 2 mL of PBS, cut up, and incubated in 37 °C water for 0.5 h. Subsequently, after removing the debris, another 2 mL of PBS was added to the container, and then, 10 µL of diluted sperm suspension was carefully dispensed onto a hemocytometer plate for counting. Another 10 µL of diluted sperm suspension was dispensed onto glass slides, allowed to dry naturally, fixed with methanol, and stained with eosin. After drying again at room temperature, the morphology of the spermatozoa was observed and imaged with an inverted microscope, and the number of normal sperm and abnormal sperm in a 400× field of view was recorded. The % of sperm malformation was then calculated by dividing the number of abnormal sperm by the total number of sperm.

### 2.3. Measurement of Serum Testosterone Levels

Serum testosterone levels were measured with an enzyme-linked immunosorbent assay (ELISA) kit (E-EL-0155c, Elabscience, Wuhan, China). Briefly, serum was added to a 96-well plate pre-coated with antigen, followed by the addition of biotinylated detection antibodies, incubation with horseradish peroxidase (HRP) conjugate, and the addition of the substrate reagent. The absorbance (OD) of all 96 wells was measured at 450 nm. Six serum samples were randomly selected from each group and tested in triplicate.

### 2.4. Histopathological Analysis

Left testis tissues were fixed with 4% paraformaldehyde for more than 24 h, followed by dehydration, embedding, and slicing into 4 µm thick sections for hematoxylin and eosin (H&E) staining. Subsequently, all the testicular sections were observed by a light microscope and quantitative analysis was performed using Fiji software. Briefly, six testes’ samples from each group were randomly chosen, six random 200× fields of view were selected for each testicular section, the number of layers of spermatogenic epithelium and damaged seminiferous tubules (ST) in each field was counted, and the average diameter of the ST in each field of view was measured.

### 2.5. TUNEL Staining

TUNEL apoptosis assay kit (E-CK-A325, Elabscience, Wuhan, China) was used for apoptosis detection. All samples were observed with a fluorescence microscope (ECLIPSE 90i, Nikon, Tokyo, Japan). The process of the experiment was performed according to the manufacturer’s instructions. Details can be found in our previous reports [29].

### 2.6. Western Blotting Analysis

Testicular tissues were homogenized in RIPA lysis buffer, and the supernatants were collected after homogenization. Subsequently, the protein concentrations were detected using a BCA kit according to the manufacturer’s instructions. Primary antibodies against STRA8 (ER1917-31, Huabio, Hangzhou, China) and SCYP3 (162171, ZENBIO, Chengdu, China) were used. Beta-Actin (EM21002, Huabio, Hangzhou, China) was used as an internal reference. Quantitative analysis was conducted by Image Lab software (version 3.0, Bio-Rad, Hercules, CA, USA).

### 2.7. Measurement of Retinol (ROL), Retinaldehyde (RE), and Retinoic Acid (RA) Levels

The concentrations of ROL (nmol/L), RE (pg/mL), and RA (pg/mL) in serum, testis, and GC-2 cells were measured using ELISA kits (YJ210350, YJ210304, and YJ210346, YUANJIE, Shanghai, China). In short, specimens, standards, and HRP-labeled detection antibodies are added sequentially to the precoated microtiter wells with ROL/RE/RA antibody, and then, the plates were incubated and thoroughly washed. Color was developed with TMB substrate. The OD was measured by a microplate reader at 450 nm. Six sample replicates and three technical replicates were set for this experiment.

### 2.8. Real-Time Quantitative Polymerase Chain Reaction (RT–qPCR)

Total RNA was extracted from testis and GC-2 cells using a Simply P Total RNA Extraction Kit (BSC52S1, Bioer, Hangzhou, China), and single-strand cDNA was then synthesized by an RT Master Mix Kit (HY-K0511, MCE). Each reaction was configured with 10 uL of reaction system: 1 uL of cDNA sample, 5 uL of SYBR Green qPCR Master Mix (HY-K0523, MCE), 0.2 uL of pre/reverse primer, and 3.6 uL of nuclease free water. A total of 3 min of initial denaturation at 95 °C, 15 s of denaturation at 95 °C (40 cycles), and 30 s of extension at 60 °C were used in the RT–qPCR programs. The relative mRNA levels of the target genes were quantified using the 2^−ΔΔCt^ method, with β-actin expression as a reference for normalization. Further details can be found in our previous reports. The primer sequences are provided in Supplementary Table S1.

### 2.9. Cell Line Culture

GC-2 cells, a mouse germ cell lines, procured from the American Tissue Culture Collection were cultured in an incubator with 5% CO_2_ and at 37 °C. DMEM (GIBICO, Grand Island, NY, USA) supplemented with 10% fetal bovine serum and 1% penicillin-streptomycin were used for cell culture, the cell was passaged at a ratio of 1:10 when it reached 80–90% confluence.

### 2.10. Cell Counting Kit-8 (CCK-8) Analysis

CCK8 assays were conducted to evaluate GC-2 cell’s vitality after DFZ exposure. GC-2 cells (5 × 10^3^ cells/well) were seeded in a 96-well plate and incubated for 24 h. Subsequently, various concentrations of DFZ (0, 10, 20, 40, 60, 80, 100, and 200 μM) were added to the plate and incubated for an additional 24 h. Then, 10 µL of CCK8 solution was added to each well and incubated at 37 °C for 2 h. OD values were measured at 450 nm.

### 2.11. Transmission Electron Microscopy (TEM)

To observe the alteration of GC-2 cell ultrastructure after DFZ exposure, we conducted TEM analysis. After 24 h of DFZ exposure, cells were collected, fixed in glutaraldehyde, and then dehydrated in a gradient of ethanol before being embedded in Epon618. Ultrathin sections were made on an ultrathin sectioning machine, then using uranyl acetate and lead citrate staining. Finally, a JEM-1400PLUS TEM was used to photograph.

### 2.12. RNA-Seq and Bioinformatics Analysis

Three samples of 0 or 20 μM DFZ-treated GC-2 cells were subjected to mRNA sequencing analysis. Differentially expressed genes (DEGs) were screened with log2 (fold change) ≥ 2 or ≤−2 and *p*-value < 0.05. We conducted a functional analysis of the DEGs using GO and KEGG enrichment analysis. Cluster analysis heatmaps were drawn by the OmicStudio tools at https://www.omicstudio.cn/tool (accessed on 26 March 2023). Metascape platforms (http://metascape.org/gp/index.html (accessed on 26 March 2023)) were adopted for DEGs functional annotation. We constructed a protein–protein interaction (PPI) network for target-enriched genes using the STRING database with an interaction score ≥ 0.4., cytoHubba plugins of Cytoscape software (Version 3.8.0, http://cytoscape.org/ (accessed on 26 March 2023)) were used to identify the top 5 Hub genes.

### 2.13. Statistical Analysis

GraphPad Prism 9.0 (GraphPad, La Jolla, CA, USA) was used for statistical analysis. Experimental data were expressed as the mean ± standard deviation. One-way ANOVA with Dunnett’s post hoc test was used to compare multiple groups, and *p* < 0.05 was considered to be statistically significant.

## 3. Results

### 3.1. DFZ Exposure Induces Testicular Injury in Mice

**Figure 1:**
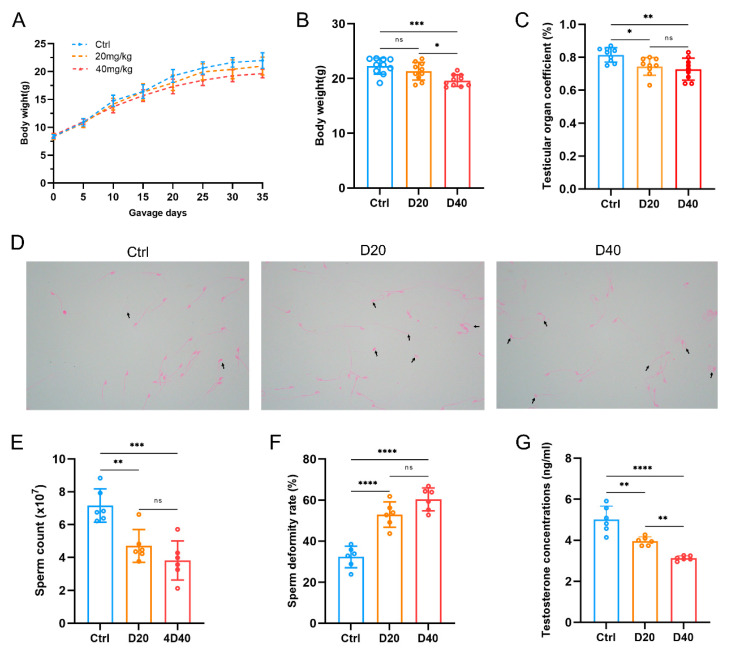
DFZ exposure induces testicular injury. (**A**) Temporal graph of body weight, (**B**) body weight at the completion of treatment, (**C**) testicular organ coefficient, (**D**) sperm morphology (black arrows denote the malformed sperm), (**E**) sperm counts, (**F**) sperm deformity rate, and (**G**) serum testosterone concentration in different groups. * *p* < 0.05, ** *p* < 0.01, *** *p* < 0.001, **** *p* < 0.0001, ns. indicates not statistically significant versus the control group.

Compared with the control group, the body weight (Figure 1A,B), testicular organ coefficients (Figure 1C), and serum testosterone levels (Figure 1G) were significantly decreased in the D20 and D40 groups. As shown in Figure 1D, the control group occasionally exhibited sperm head abnormalities, and most of the sperm had normal structures. However, the DFZ-treated group exhibited significantly more sperm abnormalities, including uncinate head abnormalities, broken tail abnormalities or coiled abnormalities, with most of the abnormalities occurring in the caudal part of the sperm. Additionally, the sperm count decreased (Figure 1E) and the sperm malformation rate (Figure 1F) increased in both D20 and D40 groups. Interestingly, while the D40 group had more pronounced decreases in body weight and testosterone levels than the D20 group, the differences in testicular organ coefficients, sperm counts, and sperm malformation rates were not statistically significant between the two groups.

**Figure 2:**
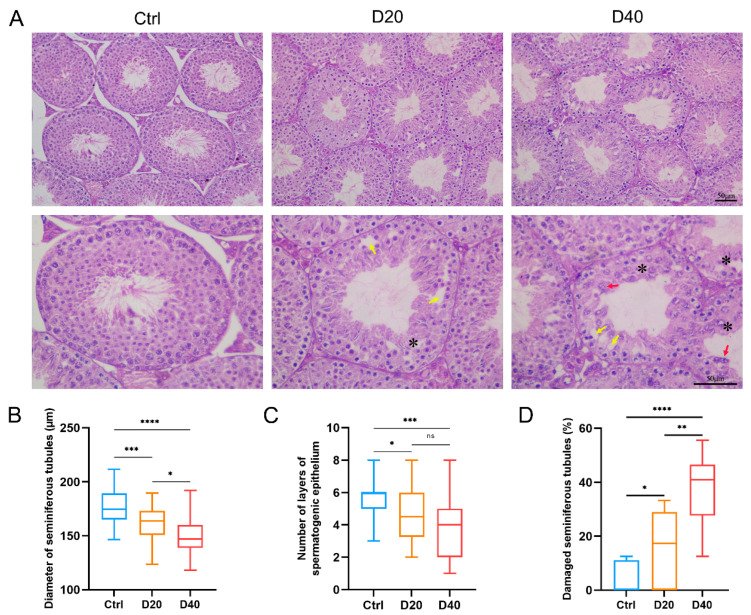
DFZ exposure leads to pathological changes in testicular tissue. (**A**) H&E staining of testicular tissue (black markers indicate the disrupted germinal epithelium. Yellow arrows indicate the vacuolation of the germinal epithelium. Red arrows indicate the sloughing of germ cells. Asterisk indicates damaged seminiferous tubules), (**B**) diameter of seminiferous tubules, (**C**) number of spermatogenic epithelium layers, and (**D**) percentage of damaged seminiferous tubules in different groups. * *p* < 0.05, ** *p* < 0.01, *** *p* < 0.001, **** *p* < 0.0001, ns. indicates not statistically significant versus the control group.

H&E staining results revealed significant histopathological changes (Figure 2A). In the DFZ-treated group, the diameter of the ST was significantly reduced, the arrangement of the germinal epithelium was disorganized, the number of layers was decreased, vacuolization was evident, and shed germinal cells were observed in the lumen of the tubules. Furthermore, the diameter of the ST (Figure 2B) and the number of the spermatogenic epithelium (Figure 2C) in the DFZ-treated groups were significantly lower than those in the control group, while the number of damaged seminiferous tubules was significantly higher than that in the control group, and a dose-dependent effect was observed (Figure 2D).

**Figure 3:**
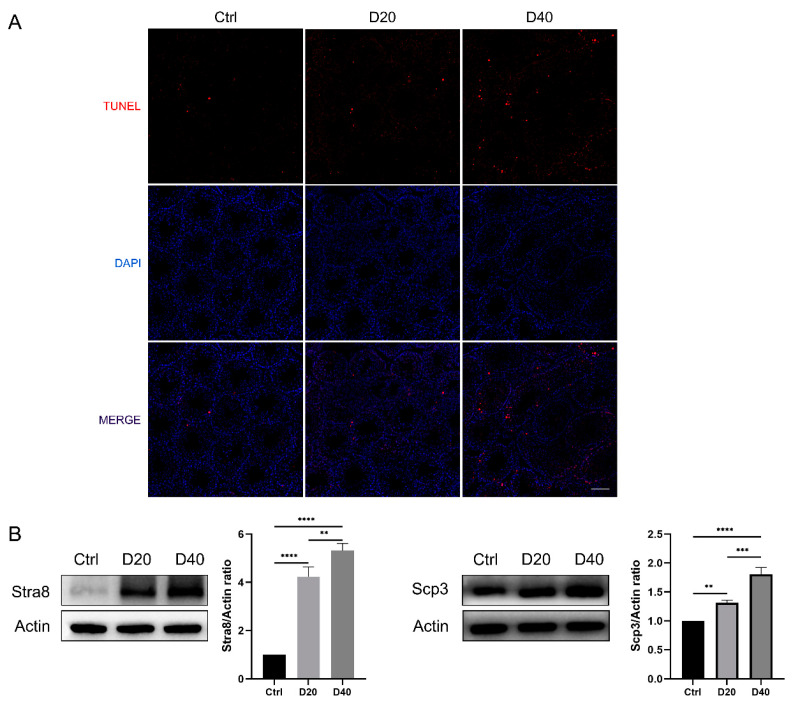
DFZ exposure induces increased apoptosis and abnormal meiosis in testicular tissues. (**A**) TUNEL staining of testicular tissue (the scale bar represents 100 µm) and (**B**) protein (Stra8 and Scp3) expression levels in different groups. ** *p* < 0.01, *** *p* < 0.001 and **** *p* < 0.0001 versus the control group.

Tunnel assay showed a significant increase in apoptosis rate in D20 and D40 groups, and apoptosis occurred mainly in spermatocytes (Figure 3A). The protein expression levels of the meiotic marker proteins STRA8 and SYCP3 rose dramatically in the D20 and D40 groups, suggesting the occurrence of abnormal meiosis in germ cells (Figure 3B).

### 3.2. DFZ Exposure Interferes with the Testicular RA Pathway

**Figure 4:**
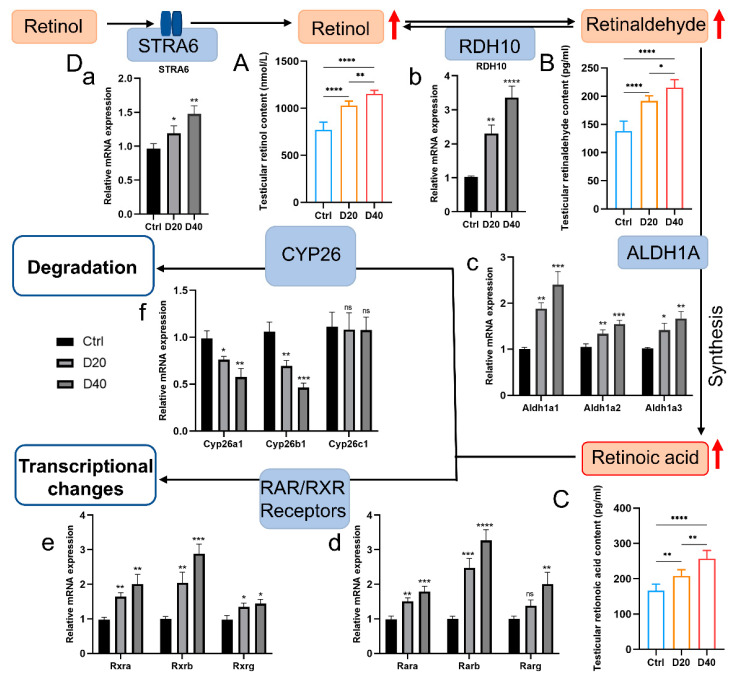
Effects of DFZ exposure on the testicular RA pathway. (**A**) RA, (**B**) RE, and (**C**) ROL concentrations in testicular tissues in different groups. (**D**) Relative mRNA expression level of key genes in RA pathway. (a–c) genes in RA synthesis, (d,e) RA receptors, and (f) genes in RA catabolism. * *p* < 0.05, ** *p* < 0.01, *** *p* < 0.001, **** *p* < 0.0001. ns. indicates not statistically significant versus the control group.

To investigate the effect of DFZ on RA pathway homeostasis, we measured the levels of RA, RE (the stored form of ROL) and ROL (the substrate of RA synthesis) in testicular tissues. The results revealed that the level of RA, RE, and ROL in testicular tissues were significantly elevated after DFZ treatment (Figure 4A–C).

RT–qPCR experiments were conducted to investigate the alteration expression of key enzymes and receptors that are involved in RA synthesis and catabolism. The mRNA expression levels of STRA6, RDH10, and ALDH1A1-3, which are key enzymes involved in ROL, RE, and RA synthesis, were significantly increased after DFZ treatment (Figure 4D (a–c)). Additionally, the mRNA expression levels of nuclear RA receptor RARa, RARb, and RARg, as well as nuclear retinoid X receptor RXRa, RXRb, and RXRg, were also increased (Figure 4D (d,e)). Furthermore, CYP128A1 and CYP26B1, which are key enzymes in RA catabolism, were significantly decreased after DFZ exposure (Figure 4D (f)), indicating that RA catabolism was inhibited. Interestingly, we noticed that the expression level of another enzyme related to RA catabolic processes, CYP26C1, did not change, likely because the inhibitory effect of DFZ on testicular RA catabolism was primarily accomplished through the inhibition of CYP26A1 and CYP26B1, which is consistent with previous reports by Rachel L Gewiss et al. [30].

### 3.3. Toxic Effects of DFZ on GC-2 Cells

**Figure 5:**
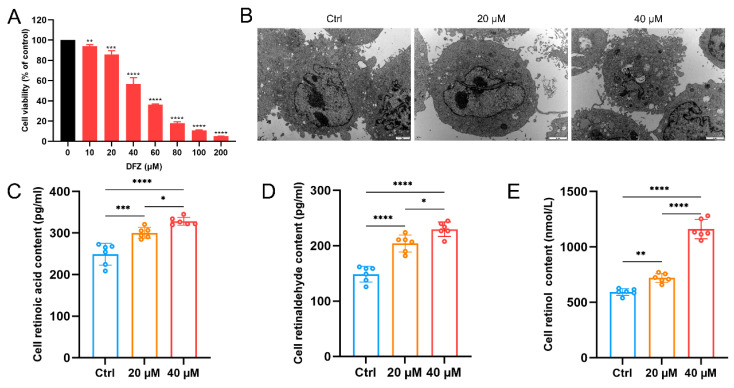
Toxic effects of DFZ on GC-2 cells. (**A**) Cell viability assay of GC-2 cell after exposure to different concentrations of DFZ for 24 h. (**B**) Altered cellular ultrastructure observed by transmission electron microscopy (the scale bar represents 2 µm). (**C**) RA, (**D**) ROL, and (**E**) RE concentrations in GC-2 cells in different groups. * *p* < 0.05, ** *p* < 0.01, *** *p* < 0.001, **** *p* < 0.0001 versus the control group.

GC-2 cells were exposed to gradient concentrations of DFZ to evaluate DFZ cytotoxicity. As a result, DFZ exposure significantly inhibited the viability of GC-2 cells with cell viability being 85.96% after GC-2 cells were treated with 20 µM DFZ for 24 h (Figure 5A), which was used for RNA sequencing. TEM images revealed that the nuclei were large and intact and the organelle structure was clear in the control group, while vacuoles in the cytoplasm and fused aggregates of organelles appeared after exposure to DFZ (Figure 5B).

We assessed the ROL, RE, and RA levels in GC-2 cells after exposure to 20 µM and 40 µM of DFZ (Figure 5C–E), and these levels were significantly increased compared with the control group, which was in agreement with the in vivo studies.

### 3.4. Bioinformatics Analysis of the Transcriptomic Data of GC-2 Cells and qPCR Validation

**Figure 6:**
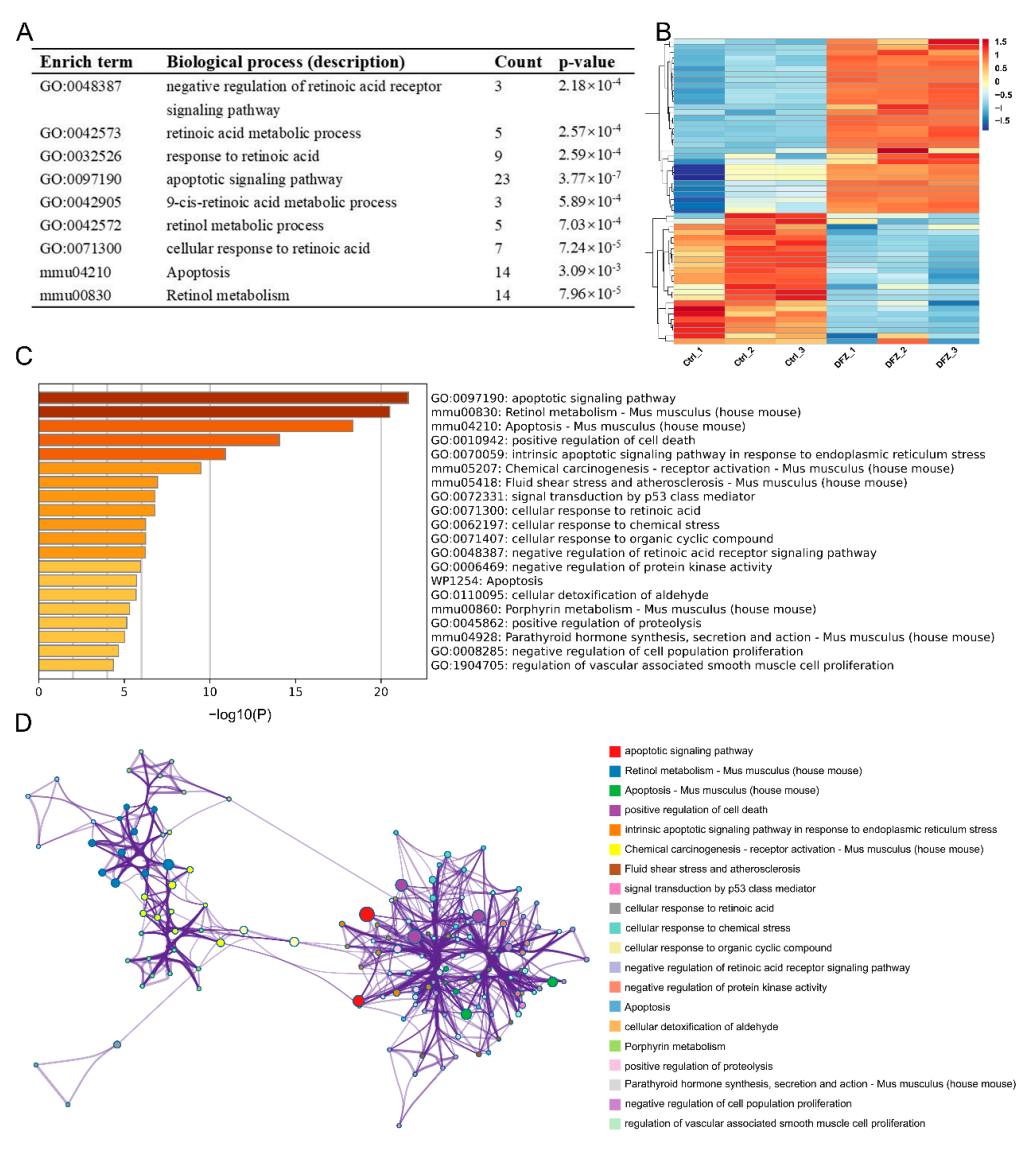
Bioinformatics analysis of the transcriptomic data of GC-2 cells in control and DFZ exposure groups. (**A**) Enriched terms related to RA pathway and apoptosis. (**B**) Heatmap, (**C**) Ontology clusters, and (**D**) network of DEGs enriched in RA pathway- and apoptosis-related terms.

DEG enrichment analysis revealed that numerous terms related to RA pathway and apoptosis process were significantly enriched in GO and KEGG enrichment analysis (Figure 6A), such as negative regulation of RA receptor signaling pathway (GO:0048387), RA metabolic process (GO:0042573), response to RA (GO:0032526), apoptotic signaling pathway (GO:0097190), 9-cis-RA metabolic process (GO:0042905), ROL metabolic process (GO:0042572), apoptosis (mmu04210), and ROL metabolism (mmu00830). A total of 56 DEGs associated with RA pathway and apoptosis were enriched (Figure 6B), including 26 DEGs related to RA pathway and 31 DEGs related to apoptosis process. CYP1B1 was enriched in both RA pathway and apoptosis process.

**Figure 7:**
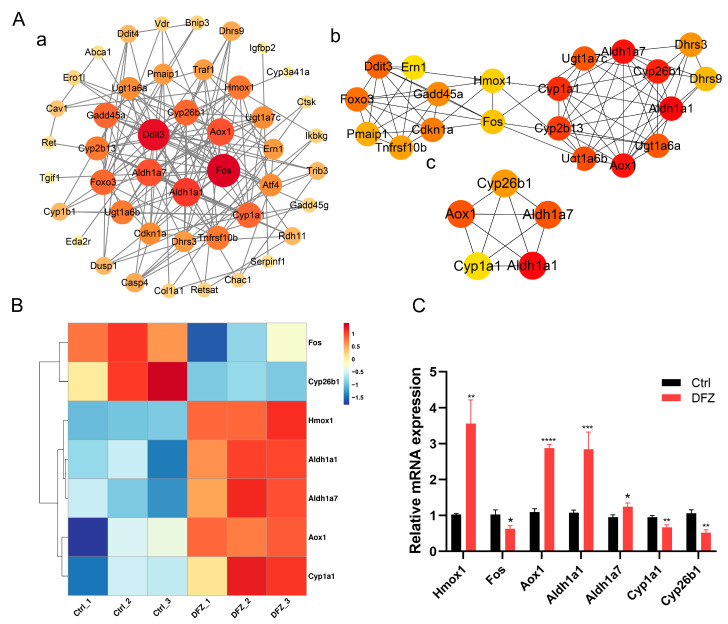
Screening and identification of key genes related to RA pathway and apoptosis. (**A**) (a,b) Protein–protein interaction (PPI) and (c) cytoHubba MCC analysis of DEGs enriched in RA pathway- and apoptosis-related terms. (**B**) Heatmap and (**C**) qPCR validation of identified key genes. * *p* < 0.05, ** *p* < 0.01, *** *p* < 0.001, **** *p* < 0.0001 versus the control group.

We submitted these 56 DEGs to the Metascape website to investigate the correlation between the enriched terms. The top 20 enriched terms sorted by *p* value are shown in Figure 6C. Our analysis revealed that these enrichment terms are interconnected and form clusters (Figure 6D). To further investigate the protein interactions among these 56 DEGs, we constructed a PPI network (Figure 7A (a,b)). ALDH1A1, ALDH1A7, AOX1, CYP26B1, and CYP1A1 were identified as hub genes using the cytoHubba MCC plugins (Figure 7A (c)). Transcriptome analysis suggested that HMOX1, AOX1, ALDH1A1, and ALDH1A7 expression was upregulated while FOS, CYP1A1, and CYP26B1 expression was downregulated (Figure 7B). RT–qPCR was used for validation (Figure 7C).

## 4. Discussion

In recent years, the levels of fungicide residues in the environment have been on the rise. DFZ has been found to be highly stable, difficult to degrade, and easily transferable due to its physicochemical properties [31], allowing it to persist in aquatic systems, soil, and plants [32]. DFZ residues in food and the environment can be absorbed by the human body [33], posing a potential risk to human health. However, assessments of the toxic effects caused by DFZ to the male reproductive system are still very limited [34].

In this study, we administered different doses of DFZ to male mice to investigate the etiological association between DFZ exposure and testicular injury. Because immature testes are more susceptible to environmental contaminants than mature testes, and toxic damages in prepubescent periods can persist into adulthood, we chose to expose the mice to DFZ at the prepubertal stage.

The significant decrease in body weight and testicular organ coefficients in the D20 and D40 groups suggested that testicular development may be impaired. The D20 and D40 groups showed a decrease in sperm count and serum testosterone levels, and a significant increase in sperm malformation, suggesting that prepubertal exposure to DFZ can lead to impaired reproductive function in adulthood. In particular, maintaining normal testicular tissue structure is essential for the male reproductive system, while DFZ exposure induces obvious histopathological changes in the testis. These findings confirmed that DFZ exposure can lead to toxic effects on the male reproductive system.

Xinyu Wu et al. [35] reported that DFZ exposure can induce apoptosis in the kidney in carp and Jiansheng Zhu et al. [21] demonstrated that DFZ exposure triggers cardiovascular apoptosis in zebrafish. Given the reduction of spermatogenic epithelial cells in testicular tissue after DFZ exposure, we conjectured that DFZ exposure could induce apoptosis in mouse testis. The increased apoptosis levels in the D20 and D40 groups supported our hypothesis. In addition, we found that apoptosis mainly occurred in germ cells.

Both STRA8 and SCYP3 have been proven to be critical in meiosis. STRA8 is an early meiosis marker and activated at both the transcriptional and translational levels by retinoic acid [36]. SYCP3 (a synaptonemal complex protein) is a later meiosis marker, germ cells of SYCP3-knockout mice die at the meiotic zygote stage [37]. We found that STRA8 and SCYP3 protein expression in testicular tissues was significantly increased after exposure to DFZ, suggesting that spermatogonia are abnormally active in meiosis. However, spermatogonia that have prematurely entered meiosis are prone to pachytene phase arrest and apoptosis, which could explain the decrease in sperm count and the increase in sperm malformation rate after DFZ exposure. Given that the expression level of the meiotic marker was abnormally upregulated and that the RA signaling pathway has been demonstrated to be a key mediator in spermatocyte meiosis and reproductive system development, we postulate that DFZ exposure may disrupt RA signaling.

RA is a lipid-soluble signaling molecule that is synthesized through a complex series of steps and is critical for mammalian reproductive system development [38,39]. ROL firstly binds to the retinol-binding protein (RBP) and then to the retinol receptor (STRA6) to be transported into cells [40]. After entering a cell, ROL binds to the retinol-binding protein (CRBP) and is subsequently converted to RE by ethanol dehydrogenase (ADH) or retinol dehydrogenase (RDH), and retinaldehyde dehydrogenase (ALDH1A1-3) catalyzes the formation of RA from RE [41]. Eventually, RA is transported to the nucleus by cellular RA-binding proteins (CRABP), where it regulates gene expression by binding to the nuclear RA receptor (RAR) and the nuclear retinoid X receptor (RXR) dimeric RAR reaction element [42]. The degradation of RA is mediated by the CYP26 family of enzymes [43].

Therefore, we investigated the levels of RA and its intermediates ROL and RE. We discovered that ROL, RE, and RA levels were considerably elevated in testicular tissues, with a significant dose-dependent effect. These findings confirmed our previous suspicions. However, it remains to be elucidated whether the augmented RA levels associated with DFZ exposure are a consequence of increased synthesis, reduced catabolism, or a combination of both, as well as which molecules are critically involved. In the follow-up experiment, we found the expression level of genes involved in RA synthesis processes, such as STRA6, RDH10, ALDH1A1-3, and RAR/RXR (a, b, g) were significantly increased while genes involved in RA catabolism, such as CYP26A1 and CYP26B1, were significantly decreased. These results indicate disruption in RA homeostasis after DFZ exposure.

GC-2 cell line, derived from immortalized mouse pachytene spermatocytes, is an important tool for studying the cellular effects of spermatocytes [44]. During the first spermatogenic cycle, the development of undifferentiated spermatogonia into differentiated A1 spermatogonia is dependent on Sertoli cell-derived RA, while the subsequent spermatogenic cycle is initiated by spermatocyte-derived RA [28,45]. Given that spermatocyte apoptosis was found to be significant in vivo experiments, and RA has been reported to regulate germ cell apoptosis-related processes [46], the GC-2 cell line was used for in vitro studies.

In vitro, DFZ induced significant cytotoxicity in GC-2 cells, with cell viability decreasing gradually with increased dose and cellular ultrastructure being altered after DFZ exposure. The increased levels of ROL, RE, and RA in GC-2 cells after DFZ exposure are consistent with the detection of ROL, RE, and RA levels in testes in vivo. Analysis of transcriptome results found that multiple terms related to RA signaling were enriched in the transcriptome sequencing analysis of GC-2 cells and were mainly associated with ROL and RA metabolic processes. Terms related to the apoptosis process were also enriched. By integrating the enriched terms, we found an indirect link between the apoptotic signaling pathway and ROL metabolism is established via chemical carcinogenesis receptor activation. PPI results suggest that HMOX1 and FOS are genes linking RA pathway-related genes and apoptosis-related genes, and ALDH1A1, ALDH1A7, AOX1, CYP26B1, and CYP1A1 were identified as hub genes. Previous studies have reported that the RA derivative increases expression of HMOX1, induces apoptosis in ARPE-19 cells [47] and RA inhibits the expression of c-FOS and induces apoptosis in rat liver [48]. ALDH1A1 and ALDH1A7 are members of the ALDH family, and AOX1 is an aldehyde oxidase, and these enzymes catalyze the production of RA from RE [49]. CYP26B1 has been widely known as a key gene in the RA catabolic process and a major target for RA metabolic abnormalities caused by TOFs [50]. CYP1A1, aryl hydrocarbon receptor-dependent genes, mediate all-trans-RA catabolism [51].

Based on the above reports and our experimental results, we conjecture that DFZ exposure could enhance RA synthesis through the upregulation of ALDH1A1, ALDH1A7, and AOX1 expression, while reducing RA catabolism through the downregulation of CYP26B1 and CYP1A1, thus leading to increased HMOX1 expression and decreased FOS expression, and eventually mediating apoptosis in GC-2 cells. The specific molecular mechanism underlying RA pathway dysregulation-induced testicular damage after DFZ exposure needs further exploration. DFZ has been reported to induce apoptosis through the induction of oxidative stress in zebrafish and SH-SY5Y cells [21,23]. Therefore, it remains to be investigated whether the apoptosis observed in testicular tissue and GC-2 cells is associated with oxidative stress injury caused by DFZ. Further studies are warranted to elucidate this potential mechanism. Moreover, to evaluate the effects of DFZ exposure on the male reproductive system throughout the whole life cycle, the toxic effects of prenatal exposure to DFZ on the reproductive system of offspring need to be further investigated. Our findings will offer a theoretical basis for studying the endocrine-disrupting effects of DFZ and help expand the understanding of the toxic effects of DFZ.

## 5. Conclusions

In our study, we found that prepubertal DFZ exposure could induce testicular injury in adulthood. In addition, DFZ could increase RA synthesis and decrease cytochrome p450-mediated RA catabolism, thus disrupting RA homeostasis in the testis. The imbalance of RA in the testis is an important cause of DFZ-induced testicular injury, possibly linked to the increased apoptosis of GC-2 cells, which warrants further investigation.

## Data Availability

The data presented in this study are available on request from the corresponding author. The data are not publicly available due to privacy.

## Supplementary Materials

The following supporting information can be downloaded at: https://www.mdpi.com/article/10.3390/toxics11040328/s1, Table S1: The sequences of the primers used for qRT-PCR.
