# Supplementary material for: Difenoconazole Exposure Induces Retinoic Acid Signaling Dysregulation and Testicular Injury in Mice Testes
Source: Toxics. 2023 Mar 30;11(4):328. doi: 10.3390/toxics11040328 (PMC10142862; doi:10.3390/toxics11040328)
Supplement: Supplementary file 1 [file toxics-11-00328-s001.zip › toxics-2280409-supplementary.pdf]

Table S1: The sequences of the primers used for qRT-PCR.

Gene	Primer Sequence (F 5′ to 3′)	(R 5′ to 3′)
Stra6	GGCCTACACATTGCTCCACA	AGGCAGGGTGATGTGGTTTT
Rdh10	AGACACGGGCATGTTCAGAG	CACACGACGGCTTCAAAAGG
Aldh1a1	AACTGTGTGGGGAGGAGTCT	TGCAAACCTCTCCCTTTGCT
Aldh1a2	TCCAACCCCACCCTCTTTCT	CAGTGCTGCCCACACCAA
Aldh1a3	ACCGGAGAGTGCGAACCA	CCGGCTGTCCGTTTTCC
Rara	GCTGGGCAAGTACACTACGA	CAGGATATCCAGGCAGGCAG
Rarb	CAGCTGGGTAAATACACCACGAA	GGGGTATACCTGGTACAAATTCTGA
Rarg	GACCCAGCCAACCCTACATG	CCCGGAGGTCGGTGATTT
Rxra	TGTTTGCAATGGTGGTGTGG	GCAGGGTGGCTAATGAGCTG
Rxrb	CAAGTGTCTGGAGCACCTGTTC	CCATGAGGAAGGTGTCAATGG
Rxrg	CGCATCCCCCACTTCTCA	CCTGCCCGGAGTAGAATGAC
Cyp26a1	CTGCGATTGAATCCTCCGGT	GCACTATAAAGCGGTCGGGA
Cyp26b1	CCCCCTATGTTGTTCTCTGGC	CATGGGCATACAGTCCTGGC
Cyp26c1	GGTGAAACGCTGCACTGGTT	CGCGGCGGGAACTGT
Hmox1	AGGCTAAGACCCCTTCCT	TGTGTTCCTCTGTCAGCATCA
Fos	GCGAGCAACTGAGAAGACT	GTTGAAACCCGAGAACATC
Aox1	TCAGTCTCTCGGCTGTTGGA	TCAAGGGCTGGGTTTATGCT
Aldh1a7	CACACTTGGCAAGGAGGACC	AACACTCAGAGGAATAACCCCG
Cyp1a1	CAGGAGAGCTGGCCCTTTA	TAAGCCTGCTCATCCTGTG
β-actin	GGCTGTATTCCCCTCCATCG	CCAGTTGGTAACAATGCCATGT
