# Supplementary figures and images for: Difenoconazole Exposure Induces Retinoic Acid Signaling Dysregulation and Testicular Injury in Mice Testes
Source: Toxics. 2023 Mar 30;11(4):328. doi: 10.3390/toxics11040328 (PMC10142862; doi:10.3390/toxics11040328)

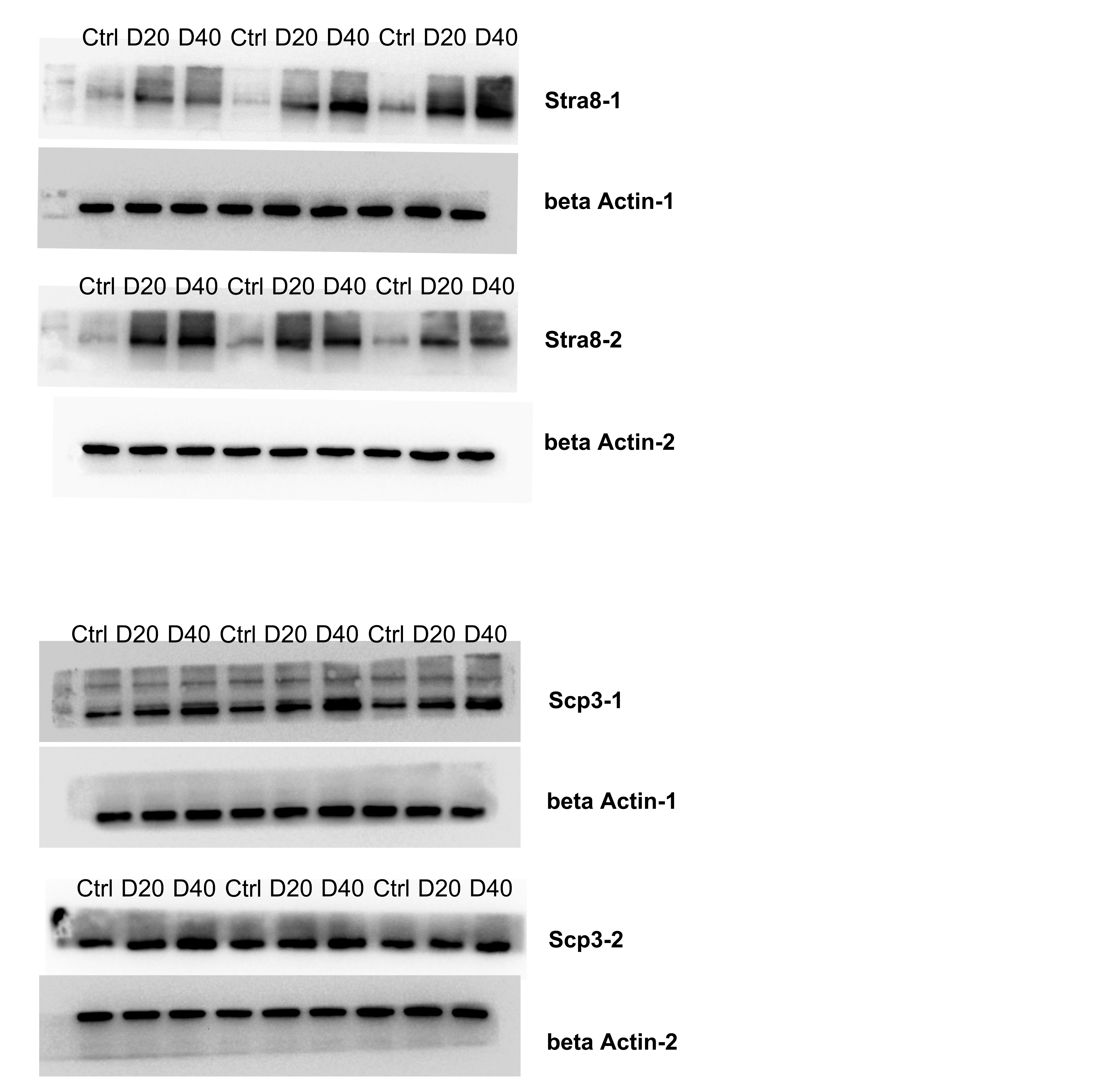

Supplement: Supplementary file 1 [file toxics-11-00328-s001.zip › Raw data of weatern blot/Stra8 WB╫Θ═╝.tif]
